# Hypernatremia in Diabetic Ketoacidosis: Rare Presentation and a Cautionary Tale

**DOI:** 10.7759/cureus.11841

**Published:** 2020-12-02

**Authors:** Gabriel Ibarra, Monil M Majmundar, Enrique Pacheco, Harshvardhan Zala, Shobhana Chaudhari

**Affiliations:** 1 Internal Medicine, New York Medical College, Metropolitan Hospital Center, New York, USA; 2 Internal Medicine, Yale Waterbury Hospital, Waterbury, USA; 3 Internal Medicine, Amidhara Hospital, Surat, IND; 4 Geriatrics, Metropolitan Hospital Center, New York, USA

**Keywords:** metabolic changes and diabetes, severe diabetic ketoacidosis, dehydration, hypernatremia

## Abstract

Hyponatremia in diabetic ketoacidosis (DKA) is common and can be due to several reasons. However, hypernatremia in DKA is rare and can be life-threatening. Its exact etiology is not clear and several mechanisms related to water deficit from inadequate oral intake and free water loss that supersedes the electrolyte loss through diarrhea or vomiting have been proposed. Treating the DKA more aggressively than the hypernatremia itself, choosing a hypoosmolar fluid, and switching to D5-0.45% saline, when glucose has decreased, are some of the vital considerations for the management of hypernatremia in DKA. We present a 44-year-old male patient with an unclear history of DKA with unusually severe hypernatremia that gradually responded to aggressive management of DKA with rigorous IV hydration and the above-mentioned strategies.

## Introduction

Diabetic ketoacidosis (DKA) causes a hyperosmolar state driven by the osmotic force of hyperglycemia in the intravascular space. Dilutional hyponatremia is common due to water driven into the intravascular space from inside cells. On rare occasions, hypernatremia is found in DKA. Hypernatremia is usually explained by a water deficit from inadequate oral intake and free water loss that supersedes the electrolyte loss through diarrhea or vomiting. This report serves as a reminder of the importance of a fluid management approach in patients with DKA with a rare manifestation of hypernatremia.

## Case presentation

A 44-year-old homeless male patient with unclear past medical history at admission was brought in by ambulance for sudden onset of intractable non-bilious, non-bloody vomiting associated with altered mental status. A fingerstick glucose screening upon admission revealed a blood glucose of >600 mg/dL. His blood pressure was 163/83 mmHg, heart rate of 92 beats per minute, respiratory rate of 18 breaths per minute, and oxygen saturation of 99% on room air. Physical examination was remarkable for a thin physique, altered sensorium, but responsive to verbal and tactile stimuli and incoherent speech. The rest of the physical examination was benign except for pinpoint pupils.

Initial laboratory data revealed a blood sugar of 875 mg/dL, pH of 7.27, lactate of 3.8 mmol/L, bicarbonate of 13 mEq/L, partial pressure of CO_2_ of 43 mmHg, sodium of 147 mEq/L (corrected sodium of 159 mEq/L), potassium of 5.1 mEq/L, chloride of 93 mEq/L, anion gap of 41 and large blood and urine ketones were detected. Additionally, the patient had an elevated BUN/creatinine of 56/2.0 mg/dL suggestive of acute kidney injury from likely pre-renal cause and urine toxicology positive for opiates, cocaine, and methadone. Due to the acuity of his altered sensorium, a head CT was ordered and revealed an old left middle cerebral artery infarction. The initial acid-base analysis revealed a high anion gap metabolic acidosis, mild respiratory acidosis from possible lack of optimal respiratory compensation due to suppression of the respiratory drive caused by opioid intoxication, and a delta-delta gap more than two suggestive of metabolic alkalosis from the recurrent vomiting (Table 1).

**Table 1:** Electrolytes changes within 24 hours

Variable/Time since admission	Hour 1	Hour 3	Hour 6	Hour 12	Hour 16	Hour 20	Hour 24
Serum Sodium (mEq/L)	147	153	151	152	154	152	150
Corrected serum sodium	159	162	156	157	158	154	151
Anion GAP	41	36	28	26	18	14	15
Potassium (mEq/L)	5.1	4.5	5.3	5.7	4.8	4.6	3.7
Chloride (mEq/L)	93	103	109	109	113	113	108
Bicarbonate (mEq/L)	13	14	14	17	23	25	27
Glucose (mg/dL)	875	689	440	412	354	226	160
pH	7.27	7.20	-	7.30	-	-	7.48
pCO_2_ (mmHg)	31.2	36.4	-	42.2	-	-	38.1
Calculated osmolality	362	363	343	342	341	328	318
Serum osmolality (mOsm/kg)	363	-	-	-	-	-	-

The patient initially received 2 liters of 0.9% saline solution. Electrolytes repeated after three hours, revealed an increase of corrected sodium from 159 mEq/L to 162 mEq/L. The fluid choice was changed to Ringer’s Lactate (LR) infusion at 200 mL/hour, and 10 units bolus of regular insulin was given and continued with insulin intravenous infusion at 5.5 units/hours adjusted at his weight (55 kg). Subsequent evaluation of electrolytes revealed a steady-state of the corrected serum sodium at 156-158 mEq/L between hour 6 to hour 20 since the first serum sodium obtained at admission.

Once the blood glucose was close to 200 mg/dL, we decided to change the fluids from LR to dextrose 5% (D5W) to initiate the correction of sodium at a rate of approximately 0.5 mEq/L/hour. After 24 hours, the corrected sodium was 151 mEq/L compared to 159 mEq/L at admission with a noticeable change of his mental status that slowly improved when the serum sodium decreased and not when the blood sugar decreased, revealing that the altered mental status was likely from the hypernatremia and not the DKA. The patient was bridged 24 hours after the initial encounter with long-acting insulin and short-acting insulin at 0.2 Units/Kg was started once the patient was able to tolerate oral intake, his mental status improved, and the anion gap resolved.

Once the patient was fully alert, he expressed that suffered from Type 1 Diabetes and had an opioid and cocaine use disorder. The patient was later downgraded to general ward and discharged after counseling regarding risks of opioid and cocaine use, especially in the context of Type 1 diabetes.

## Discussion

Here, we describe a case of a patient with DKA and hypernatremia. Hypernatremia, although an unusual finding in adults with DKA, has been more commonly described in the pediatric population and is usually associated with excessive soft drink ingestion [1]. In DKA, we expect to find normal or low serum sodium due to the dilutional effect of hyperosmolar status caused by elevated blood glucose that shifts water from the intracellular space to the extracellular space. The osmotic force of glycosuria that drives the sodium by osmotic diuresis contributes to a volume depletion status. The causes of hypernatremia in DKA could potentially be explained by excessive water losses relative to the osmotic loss of sodium through the urine. Additionally, recurrent vomiting, which is classically seen in patients with DKA, can exacerbate the excessive volume loss.

There is not a clear approach to fluid management of hypernatremia in DKA. In a patient with low or normal serum and DKA, normal saline is the fluid of choice [2]. Normal saline will cause intravascular expansion and correct the hyperosmolar hypovolemic hyponatremia seen in these patients. This is based on the consideration that every liter of normal saline contains 154 mmol per liter and can, theoretically, increase serum sodium by 0.41mEq/L per liter of normal saline given, assuming serum sodium of 140 mEq/L and total body water of 60%. However, in patients with hypernatremia and DKA, we do not want to increase the serum sodium. Hence, solutions with less sodium content, such as LR (130 mmol per L of Na for every liter of the solution infused) or half normal saline (77 mmol per L of Na for every liter of the solution infused) are more appropriate to decrease the serum sodium at an initial stage.

The rate of sodium correction is another critical variable. The standard goal to correct hypernatremia is at 10 mEq/L per 24 hours [3]. However, the evidence on the correction rate of acute hypernatremia is not as robust as it is for acute hyponatremia. Inadequate information on the rate of correction of hypernatremia underlies the importance of recognizing that hypernatremia in DKA warrants a careful selection of the type of fluid used.

**Figure 1:**
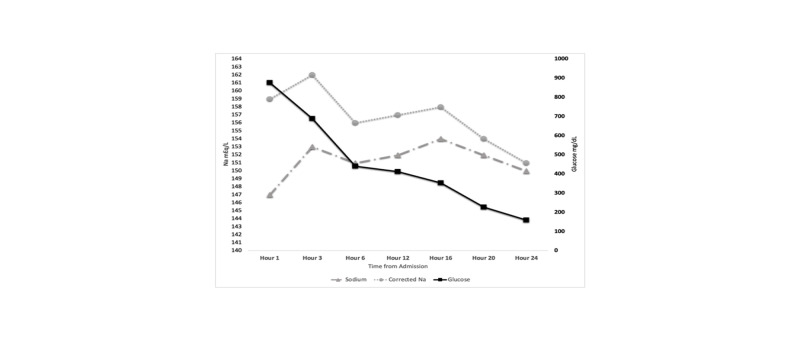
Trend of sodium and glucose over a 24-hour period

In our case, we decided to manage the patient with LR for the first 6 hours after we observed that 2 liters of normal saline increased the corrected serum sodium from 159 mEq/L to 162 mEq. The LR allowed us to generate a slower decrease in serum sodium compared to a potential more rapid decrease that half normal saline could have caused in serum sodium. Later, we observed that continuous LR, maintained a temporary steady state of serum sodium from hour 6 to hour 16 until the blood glucose was driven to a level that allows us to correct further the hyperosmolality caused by the hypernatremia and decrease the risk of cerebral edema (Figure 1).

Half-normal saline would have also been a good choice. Half normal saline could have decreased the serum sodium per every liter of half saline by 2.4 mEq/L. The patient had a water deficit of 4.1 liters (calculated by free water deficit =%total body water*weight (0.6 for our patient)*(current Na/Desired Na-1)), requiring approximately 6-7 liters of half-normal saline (accounting for insensible loss) to achieve our goal of free water deficit replenishment. Although this amount of half-normal saline could have decreased sodium at a significantly faster rate (approximately 14-18 mEq/L). So faster rate of sodium correction with a regular decrease in glucose, could have decreased the serum osmolality drastically, leading to cerebral edema. D5W (0 mmol per L), which is the most hypotonic of these solutions, would not be the initial appropriate fluid since it could have further increased the blood sugar and decreased the serum sodium even faster. After we achieved a blood glucose of approximately 354 mg/dL. We later changed to D5W and allowed us to smoothly decreased the sodium to around 150 mEq/L during the next eight hours. Although 354 mg/dL was an unusually elevated blood sugar to transition to D5W, since it is normally expected to switch to D5 solutions around a blood sugar of 250 mg/dL, we decided to change from LR to D5W and increase the insulin infusion rate to start correcting the serum sodium. This decision was made once the corrected sodium plateau at 158 mEq/L, the pH was closer to 7.4, bicarbonate was 23 mEq/L and the mental status of the patient had minimally changed at that point, suggesting the altered mental status was driven primarily by the hypernatremia.

Another important consideration is the order in which we treat these two hyperosmolar states. DKA is more life-threatening than hypernatremia. Low pH can cause increased proteolysis [4] and an inability of the proteins to function at their physiological pH dysregulating the normal function of cells [5]. It can also decrease systemic responsiveness to catecholamines, leading to hypotension, organ dysfunction, and death if left untreated. In moderate to severe hypovolemic hyperosmolar hypernatremia, what warrants rapid management is dehydration. This is why it would may be more critical to treat DKA first and correct hypernatremia later. In our case, we prioritize the treatment of DKA and later decide to concomitantly correct the hypernatremia once the blood glucose was in a more acceptable range, preventing a rapid osmolality change that could have led to brain edema. This highlights the importance of the order in which we treat hypernatremia in DKA.

We also considered the leading cause of the patient’s altered mental status. Moreover, the clinical sign that DKA and hypovolemic hypernatremia have in common is altered sensorium. What causes altered mental status in a patient with DKA is less clear than what causes it in hypernatremia. A retrospective analysis of 216 patients with DKA, revealed that a low pH was an independent factor associated with altered mental status and was synergistic with high osmolality [6]. However, altered sensorium was previously attributed to the hyperosmolar status of the intravascular space that decreases the water content in the brain [7]. In hypernatremia, what causes altered sensorium is the cellular dehydration that the osmotic drive sodium has shifting water from inside to outside the brain.

In our patient, there were two potential causes for altered sensorium. First, the acidosis caused by the blood ketones, and second, acute hypernatremia leading to a dual source of hyperosmolality from the elevated blood glucose and serum sodium. Although opioid intoxication was potential confounder of the altered mental status, the improvement in the patient mentation was directly correlated with the sodium level correction, not the blood glucose level correction. It is also relevant to note that the pH may have not been as low as expected due to the concomitant metabolic alkalosis driven by the episodes of emesis prior to admission, perhaps preventing a worsening metabolic acidosis, although was likely an additional contributor for the hypernatremia. We also considered that cocaine use, a potent Renin-Angiotensin-Aldosterone System activator, may have further contributed to the urinary reabsorption of sodium in parallel with the water loss from the osmotic diuresis driven by glycosuria [8].

## Conclusions

This case report highlights three vital considerations when treating patients with hypernatremia and DKA. First, the decision to treat DKA more aggressively than the hypernatremia due to the intrinsic life-threatening risk of DKA. Secondly, at the initial stage, choosing hypoosmolar fluid that contains sodium close to a lower limit of normal serum sodium (such as Ringer’s Lactate as compared to half-normal saline) to allow glucose decrease first, at the same time maintaining serum sodium at steady-state and not increasing. Third, one should switch to D5W or D5-0.45% saline, when glucose has decreased, to slowly decrease serum sodium that avoids a rapid shift in plasma osmolality and gives the brain time to adapt, decreasing the risk of cerebral edema. This case report highlights the importance of understanding the management approach required for hypernatremia and DKA to prevent complications associated with these two conditions.
